# Supplementary material for: Baseline Characterization of the Gut Microbiota of Field and Colony Populations of Phlebotomus tobbi and Preliminary Assessment of the Anti-Leishmanial Activity of Cultivable Bacteria
Source: Pathogens. 2026 Jun 23;15(7):658. doi: 10.3390/pathogens15070658 (PMC13416292; doi:10.3390/pathogens15070658)
Supplement: Supplementary file 1 [file pathogens-15-00658-s001.zip › pathogens-4357168-File S1/Composition_Barplots/Figures/charts/I0txNzReB7X1lGz0qxwJIqSmGFDyua_legend.pdf]

k__Bacteria;p__Actinobacteria;c__Actinobacteria;o__Actinopolysporales
k__Bacteria;p__Actinobacteria;c__Actinobacteria;o__Corynebacteriales
k__Bacteria;p__Actinobacteria;c__Actinobacteria;o__Micrococcales
k__Bacteria;p__Actinobacteria;c__Actinobacteria;o__Propionibacteriales
k__Bacteria;p__Actinobacteria;c__Actinobacteria;o__Pseudonocardiales
k__Bacteria;p__Firmicutes;c__Bacilli;o__Bacillales
k__Bacteria;p__Firmicutes;c__Bacilli;o__Lactobacillales
k__Bacteria;p__Firmicutes;c__Clostridia;o__Clostridiales
k__Bacteria;p__Proteobacteria;c__Alphaproteobacteria;o__Rhizobiales
k__Bacteria;p__Proteobacteria;c__Alphaproteobacteria;o__Rhodospirillales
k__Bacteria;p__Proteobacteria;c__Alphaproteobacteria;o__Rickettsiales
k__Bacteria;p__Proteobacteria;c__Alphaproteobacteria;o__Sphingomonadales
k__Bacteria;p__Proteobacteria;c__Betaproteobacteria;o__Burkholderiales
k__Bacteria;p__Proteobacteria;c__Gammaproteobacteria;o__Enterobacteriales
k__Bacteria;p__Proteobacteria;c__Gammaproteobacteria;o__Oceanospirillales
k__Bacteria;p__Proteobacteria;c__Gammaproteobacteria;o__Pseudomonadales
k__Bacteria;p__Proteobacteria;c__Gammaproteobacteria;o__Xanthomonadales
